# Descriptive study on psychological first aid for COVID-19 patients’ relatives

**DOI:** 10.1192/j.eurpsy.2021.1760

**Published:** 2021-08-13

**Authors:** M. García Moreno, A. De Cós Milas, L. Beatobe Carreño, M.E. Pelayo Delgado, M.T. González Salvador, R. De Arce Cordón

**Affiliations:** 1 Psychiatry, HOSPITAL UNIVERSITARIO PUERTA DE HIERRO MAJADAHONDA, MADRID, Spain; 2 Psychiatry, HOSPITAL UNIVERSITARIO DE MÓSTOLES, MADRID, Spain

**Keywords:** COVID-19, psychological first aid

## Abstract

**Introduction:**

The presence of psychological impact on relatives of patients admitted for Covid-19 has been described. The effectiveness of psychological first aid in critical situations has also been described. The first psychological aid describes a human response supporting another person who is suffering. This intervention is indicated for those affected by a traumatic event.

**Objectives:**

To present a theoretical review about psychological first aid and to describe data about it on relatives of patients admitted for Covid-19.

**Methods:**

Literature review about psychological first aid and data description of telephone intervention carried out by mental health professionals on family members of patients admitted for Covid-19.

**Results:**

From an initial pool of 77 Covid-19 patients, 50 were selected as telephone contact with relatives was possible. Mean age was 68.9 years, 13 were female and 37 male. 90% were admitted in internal medicine department and 10% in intensive care unit. First telephone intervention in all cases was to introduce the psychiatrist in charge of the follow-up and provide contact number of psychiatry department. In 13 relatives` support, emotional ventilation and active listening was provided and 2 of them also received therapeutic guidelines. Further phone contact was required in 12 relatives. In follow-up phone calls, all relatives received therapeutic guidelines and 3 was referred to our outpatient clinic.

**Conclusions:**

Family members of patients admitted for covid-19 may present emotional symptoms, many of them normal reactions in context of a crisis situation. A large percentage do not require a structured psychotherapeutic intervention but can benefit from a first psychological help.

**Disclosure:**

No significant relationships.

